# Inhibition of Peroxisome Proliferator-Activated Receptor Gamma Prevents the Melanogenesis in Murine B16/F10 Melanoma Cells

**DOI:** 10.1155/2014/695797

**Published:** 2014-08-28

**Authors:** Jiun-Han Chen, Junn-Liang Chang, Pei-Ru Chen, Yun-Ju Chuang, Shih-Tsang Tang, Shwu-Fen Pan, Tzer-Bin Lin, Kang-Hua Chen, Mei-Jung Chen

**Affiliations:** ^1^Department of Medical Laboratory Science and Biotechnology, College of Medical Technology, Nursing and Wellbeing, Yuanpei University, No. 306 Yuan-Pei Street, Hsinchu 30015, Taiwan; ^2^Department of Pathology & Laboratory Medicine, Taoyuan Armed Forces General Hospital, Taoyuan 32551, Taiwan; ^3^Department of Biomedical Engineering, School of Health, Ming Chuan University, No. 5 De-Ming Road, Gui Shan District, Taoyuan County, Taoyuan 333, Taiwan; ^4^Department of Biotechnology, School of Health, Ming Chuan University, Taoyuan 333, Taiwan; ^5^Department of Physiology, College of Medicine, Taipei Medical University, No. 250 Wuxing Street, Taipei 110, Taiwan; ^6^Department of Surgery, Cheng Hsin General Hospital, No. 45 Cheng Hsin Street, Pai Tou, Taipei 112, Taiwan

## Abstract

The purpose of this study was to investigate if PPAR*γ* plays a role in the melanogenesis. B16/F10 cells were divided into five groups: control, melanin stimulating hormone (*α*-MSH), *α*-MSH+retinol, *α*-MSH+GW9662 (PPAR*γ* antagonist), and GW9662. Cells in the control group were cultured in the Dulbecco's modified Eagle's medium (DMEM) for 48 hrs. To initiate the melanogenesis, cells in all *α*-MSH groups were cultured in medium containing *α*-MSH (10 nM) for 48 hrs. Cells were treated simultaneously with retinol (5 *μ*M) in the *α*-MSH+retinol group. Instead of retinol, GW9662 (10 *μ*M) was cocultured in the *α*-MSH+GW9662 group. Cells in the final group were cultured in the DMEM with GW9662. All the analyses were carried out 48 hours after treatments. The *α*-MSH was able to increase cell number, melanin production, and the activity of tyrosinase, the limiting enzyme in melanogenesis. These *α*-MSH-induced changes were prevented either by retinol or by GW9662. Further analyses of the activities of antioxidant enzymes including glutathione, catalase, and the superoxide dismutase (SOD) showed that *α*-MSH treatment raised the activity of SOD which was dependent on PPAR*γ* level. According to our results, the *α*-MSH-induced melanogenesis was PPAR*γ* dependent, which also modulated the expression of SOD.

## 1. Introduction 

Melanocytes distribute in many organs in human beings, such as nervous system, heart, iris, and epidermis [1–3]. They all originate from neural crest cells during embryonic period [4]. One of the functions in melanocytes is synthesis of melanin, indicating melanogenesis. The major rate-limited steps in the melanogenesis are catalyzed by tyrosinase (monophenol, dihydroxy-L-phenylalanine: oxygen oxidoreductase: EC 1.14.18.1) [5]. The gene encoding tyrosinase maps at chromosome 11q14-q21 in humans and chromosome 7 in mice [6]. The tyrosinase controls the initial two distinct reactions of the melanin formation process, namely, the hydroxylation of L-tyrosine to L-3,4-dihydroxyphenylalanine (DOPA) and the subsequent oxidation of DOPA to dopaquinone [7]. Two main types of melanin, eumelanin and pheomelanin, are identified in human beings physically [8]. The amounts, ratios, and types of melanin determine the skin color, and they also play the major photoprotective roles against the harmful effects by ultraviolet (UV) radiation of sunlight, including UVA and UVB [9]. Animal studies demonstrate that these melanin prevent skin from edema [10], erythema, hyperpigmentation, and inflammation [9] caused by UV exposure.

Several factors secreted from keratinocytes, fibroblasts, and even melanocytes are able to modulate the melanogenesis. For example, proinflammatory factors, IL-1α/*β*, and hormones, ACTH as well as α-melanocyte stimulating hormone (α-MSH), induce melanogenesis. Instead, endothelin 1, nitric oxide, and nerve growth factor inhibit this process [11]. The most important function of endogenous melanin is absorbing the high energy to prevent penetration of UV radiation, meanwhile scavenging the reactive oxygen species (ROS) burst by UV radiation [12–14]. Subsequently, the bursting accumulation of ROS causes inflammation in epidermis immediately, and also trigger the formation of melanin [8]. Therefore, lots of antioxidants are used to prevent the inflammation by UV exposure [9, 15, 16]. They limit the accumulation and production of ROS in epidermis.

Peroxisome proliferators-activated receptors (PPARs) were discovered in mouse liver by Issemann and Green in 1990 [17]. They are nuclear hormone receptors and are divided into three subtypes, namely, PPARα, PPAR*β*/*δ*, and PPAR*γ*. PPAR*γ* expressed in a variety of cell types, including adipocytes, macrophages, vascular smooth muscle cells, and endothelial cells [12–14]. Previous studies focus on the relationships among PPARs, lipid metabolism, and homeostasis [18, 19]. Recent studies showed that PPAR*γ* activation was able to regulate inflammatory responses, cellular proliferation, differentiation, and apoptosis [20]. The highly selective agonist for PPAR*γ*, rosiglitazone (BRL49653), belongs to structure of thiazolidinediones (TZD) and is wildly used as hypoglycemia medicine in clinic [20]. Recent study proved that the production of ROS in macrophages was significantly inhibited by TZD administration, indicating the anti-inflammation by activation of PPAR*γ* [21]. Supporting results were also observed by Jiang et al. [22] that the levels of inflammatory mediators were attenuated by TZD treatment. In our previous study, activation of PPAR*γ* reduced the severity of inflammation and the amount of ROS in pulmonary circulation [23]. Accordingly, it arose the possibility that the expression of PPAR*γ* played an important role in the melanogenesis, which responded to kinds of inflammatory conditions. Therefore, the purpose of this study was to investigate whether PPAR*γ* play a key role in the melanogenesis. According to results in the present study, we proved that the melanogenesis was through the PPAR*γ*-dependent pathway, which was in turn compensation to the expression of antioxidant enzymes.

## 2. Materials and Methods

### 2.1. Materials

The B16/F10 cell line was purchased from the Culture Collection and Research Center (Hsinchu, Taiwan); Dulbecco's modified Eagle's medium (DMEM), fetal bovin serum (FBS), phosphate buffer saline (PBS), trypsin-EDTA (TEG), albumin, Bio-Rad protein assays, melanin, L-DOPA, lysis buffer, α-MSH, retinol, and GW9662 were purchased from Sigma company (USA); the catalase assay kit, glutathione assay kit, nuclear extraction kit, PPAR*γ* transcription factor assay kit, and the superoxide dismutase assay kit were purchased from the Cayman Company (USA).

### 2.2. Cell Culture

The B16/F10 cells, 4 × 10^5^ cells, were seeded at 100 mm diameter of culture dishes containing 7 mL of DMEM with 10% FBS. Cells were then cultured at 37°C in a humid atmosphere containing 5% CO_2_. When cells reached 80% confluence, they were changed to the fresh medium and treated with agents as designed for forty-eight hours. There were five groups in this study: control, α-MSH, α-MSH+retinol, α-MSH+GW9662, and GW9662. Cells in the control group were cultured in the 7 mL of DMEM. For stimulating the melanin synthesis, the α-MSH was added to the culture medium as the final concentration as 10 nM. In the third group, cells grew in the DMEM containing 10 nM of α-MSH and 5 *μ*M of retinol. Cells in the α-MSH+GW9662 group accepted the treatments of α-MSH and GW9662 (10 *μ*M). In the last group, cells were treated only with the GW9662 as the final concentration of 10 *μ*M. All the cells grew for 48 hours in the incubator (ULTIMA, REVCO) of the environment at 37°C, a humid atmosphere with 5% CO_2_. The cell counts, levels of melanin, the activities of tyrosinase, glutathione, catalase, and superoxide dismutase were further detected in five groups. The PPAR*γ* levels in nucleus were measured in the control, α-MSH, and α-MSH+retinol groups.

Cell morphology was observed under microscopy after treatments. Then the cells were resuspended by TEG for five minutes. After dying with trypan blue, the number of cells in the dish was counted under the microscopy (LeicaDMIL Leica, Leica) in 40x field.

### 2.3. The Tyrosinase Activity Assays

The tyrosinase activity was determined by the methods of Buscà et al. [24] with minor modification. The B16/F10 cells were deattached by 0.25% trypsin-EDTA (ethylenediaminetetraacetic acid). After washing with PBS and centrifugation at 600 g, 4°C, for 4 minutes, the cell pellets were resuspended in 500 *μ*L of lysis buffer (containing 0.5% Triton X-100 (w/v), 0.1 M PMSF (phenyl methyl sulfonyl fluoride) in PBS) for 30 minutes in ice bath. The lysate was centrifuged (10,000 ×g, 20 minutes, 4°C) in an Eppendorf Biofuge. Finally, the supernatant was collected to analyze the tyrosinase activity and melanin level. The total protein concentration in each sample was determined by Bio-Rad protein assay kit. Ten microliter of each sample was transferred into the 96-well plate. The albumin was used to establish the standard curve. Ten minutes after adding the protein assay solution, the optical absorbance at 620 nm was measured by the spectrometer (V-630 Bio, Biotek). For analyzing the tyrosinase activity, 30 *μ*L of sample was mixed with 170 *μ*L of L-DOPA in the final concentration of 0.85 g/*μ*L, and then the mixture was further incubated at 37°C for 60 minutes. PBS (30 *μ*L) was added to the blank instead of the sample. The optical absorbance at 450 nm was read using the spectrophotometer (Power Wave XS, Biotek). The absorbance difference between the sample and blank (ΔA450) was used to express the amount of product, dopaquinone. The unit of tyrosinase activity was defined as the relative amount of dopaquinone catalyzed by tyrosinase within one hour in solution containing 1 gram of total proteins. Considering the total protein differences in each sample, therefore, the unit of tyrosinase activity was determined as ΔA450/(g × hr). Each sample was detected in triplicate repeat.

### 2.4. Determination of Melanin Content

Melanin content was determined by the method of Buscà et al. [24] with a few modifications. The cell pellets were dissolved in 0.5 mL of 1 N NaOH at 100°C for 30 min, then determined cell counts, and transferred 30 *μ*L to the well of 96-well plate. The 170 *μ*L of L-DOPA (0.001 g/mL) was mixed to each well to incubate together for 5 min. The melanin content was measured by the optical absorbance at 450 nm and compared with a standard curve generated by melanin with known concentrations in 1 N NaOH (Sigma Chemical Co., St. Louis, MO). The amount of melanin was further normalized by cell counts obtained previously and expressed as g melanin/cell. Each sample was measured in duplicate.

### 2.5. Measurement of PPAR*γ* Level

The measurement of PPAR*γ* was carried out following extraction of nuclear proteins. The nuclear extraction kit and the PPAR*γ* transcription factor assay kit were used in this measurement. Cells in 10 cm diameter of culture dish were suspended by 1 mL of TEG. Centrifuging to collect the cell pallet, 500 *μ*L of hypotonic buffer (0.5% Triton X-100 (w/v), 0.1 M PMSF (phenyl methyl sulfonyl fluoride) in PBS) was added for 15 minutes in ice bath. Then, the Nonidet P-40 (10%, 50 *μ*L) was added to the mixture. The cell pallet was collected by centrifuging (14,000 ×g, 30 sec, 4°C). It was further mixed with 50 *μ*L of extraction buffer. Samples were prepared as repeated six times of shaker for 30 seconds and ice bath for 10 minutes. The supernatant containing nuclear extraction was collected by centrifuging (14,000 ×g, 10 min, 4°C). The sample, 10 *μ*L, was transferred to the 96-well plate. The transcription factor binding assay buffer 90 *μ*L and the competitor ds DNA 80 *μ*L were further mixed well within the sample. The 100 *μ*L of competitor ds DNA was used as the blank. After incubating for 24 hours then washing out five times, the primary antibody to PPAR*γ* 100 *μ*L was added then incubated in room temperature for one hour. After washing out for five times, the mouse antigoat HRP conjugate, 100 *μ*L, was added to incubate for one hour in room temperature. After washing out again, the developing solution of 100 *μ*L was added to incubate for 30 minutes then the stop solution was followed to each well. The optical absorbance at 450 nm was read using the spectrophotometer (Biotek). The absorbance difference between the sample and blank was normalized to the cell counts. Each sample was detected in duplicate repeat.

### 2.6. The Glutathione Activity Assay

The total glutathione was measured by the commercial assay kit from Cayman Chemical (USA). After removing the culture medium, cells in 10 cm diameter dish were resuspended by 1 mL of TEG. Fresh medium (0.5 mL) was added and mixed well, then the cells were transferred to a new eppendorf to be centrifuged by 3000 ×g for 10 min at 4°C. Cold buffer (1 mL) was further added to the pallet containing MES and 1 mM EDTA, pH 6~7, to homogenize cells. The suspension was separated from the test sample by centrifuging in 10000 g for 15 min at 4°C. The sample was added the equal volume of* m*-phosphoric acid containing solution to remove extra proteins. After 5 minutes of incubation in room temperature, the suspension was removed by centrifuging at 3000 g for 3 min at 4°C. Each sample was mixed with 50 *μ*L of 4 M of TEAM. The 50 *μ*L of the mixture was then transferred to the well of 96-well plate. The mixture (containing MES buffer, cofactor mixture, enzyme mixture, DTNB, and Q water) was then added for 150 *μ*L to each well. This 96-well plate was then incubated in dark and shook on an orbital shaker. The absorbance was measured at 410 nm when the sample had been incubated for 25 min. The amount of GSH was calculated with the absorbance fitted to the formula established by standards. Each sample and standard was tested in duplicate.

### 2.7. The Superoxide Dismutase (SOD) Activity Assay

The SOD activity was determined based on the production of O_2_^−^ anions by the xanthine/xanthine oxidase system using a commercial assay kit (Cayman Chemical, USA). After removing the medium by suction, cells were washed with 2 mL of PBS and then 1.5 mL of fresh medium was added with TEG to culture dish. Cells were collected by centrifuging after discarding medium; the cells were rinsed with 2 mL of PBS, pH 7.4; the aforementioned process was repeated again. Cells were collected as pellet by centrifuging and further homogenized with 1 mL of cold HEPES buffer, pH 7.2, containing 1 mM EDTA, 210 mM mannitol, and 70 mM sucrose. The homogenizing tissue was centrifuged at 1,500 g for 5 min at 4°C. The supernatant was removed into a new eppendorf as sample. Each well in the 96-well plate containing 200 *μ*L of the diluted Radical Detector was added 10 *μ*L of sample or standard. The 96-well plate was then incubated for 20 minutes at room temperature when 20 *μ*L of diluted xanthine oxidase had been added. The absorbance was detected at 450 nm using a plate reader finally. The values of standards' absorbance were used to establish the standard curve and formula between absorbance and amounts of product by SOD catalyzing. The values of each sample were fitted to the formula to obtain the amount of product by SOD catalyzing. Finally, the SOD activity was expressed in U/mL. Each sample and standard was tested in triplicate.

### 2.8. The Catalase Activity Assay

The catalase activity assay was measured by the kit purchased from the Cayman Chemicals. After suction to remove the medium, 2 mL of fresh PBS was added to wash cells and then discarded again. To suspend cells, the 1.5 mL of fresh medium with TEG was then added to culture dish. The cells were then collected by centrifuging. After discarded supernatant, cells were then rinsed with 1.5 mL of PBS, pH 7.4, twice and then collected as pellet by centrifuging. To homogenize cells, the pellet was treated with 1 mL of cold HEPES buffer, pH 7.2. The homogenized cells were then collected as the suspension by centrifuging at 10,000 g for 15 min at 4°C for assay sample. Series concentrations of formaldehyde were prepared well previous as the standards. Each well was added 100 *μ*L of diluted assay buffer, 30 *μ*L of methanol, and 20 *μ*L of standards or samples. The components as positive control were 100 *μ*L of assay buffer, 30 *μ*L of methanol, and 20 *μ*L of CAT. The hydrogen peroxide, 20 *μ*L, was further added to each well to initiate the reaction. The plate was kept in dark to incubate on a shaker for 20 min at room temperature. Potassium hydroxide, 20 *μ*L, was used to terminate the reaction. Catalase Purpald (30 *μ*L) was then added as the chromogen and incubated for 10 min at room temperature. The Catalase Potassium Periodate (10 *μ*L) was then added to each well and incubated together for 5 min at room temperature on a shaker. The absorbance was detected at 540 nm. The linear relationship between the absorbance and concentration was established by standards to calculate the catalase activity of each sample. The catalase activity was expressed in nmol/min/mL. Each sample and standard was tested in duplicate.

### 2.9. Statistical Analysis

Data were presented as means±SEM. Evaluations of parameters were carried out by one-way analysis of variance. Subsequently, significant differences between any two groups were established using the Newman-Keuls multiple group comparisons. Differences were regarded as significant if *P* < 0.05.

## 3. Results

**Figure 1:**
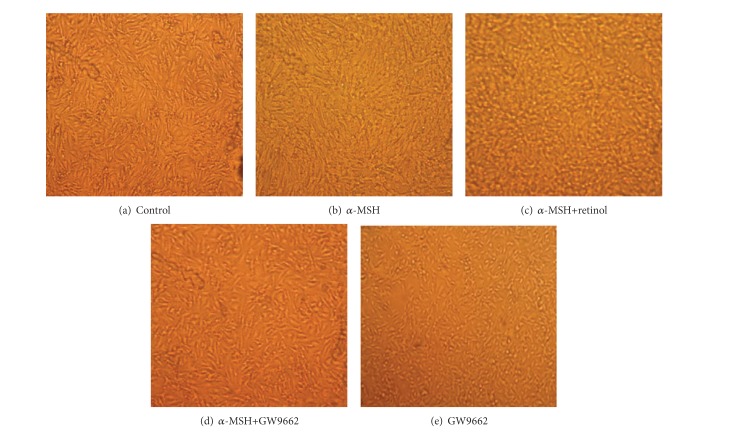
Images of the 5 groups of melanocytes; fixed magnification power of ×40. (a) Control; (b) melanin stimulating hormone (α-MSH); (c) α-MSH+retinol; (d) α-MSH+GW9662; and (e) GW9662.

**Figure 2:**
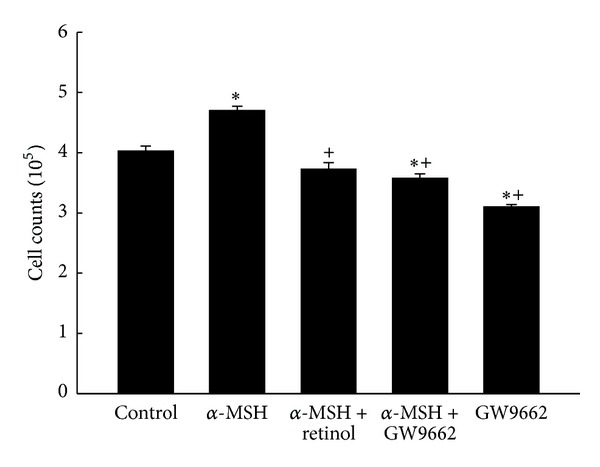
Cell counts in 5 groups. *Significant differences (*P* < 0.05) compared with the control group. ^#^Significant differences (*P* < 0.05) compared with the α-MSH group.

The morphology (Figure 1) and cell counts (Figure 2) of B16/F10 cells after treatment in each group are shown. The morphology of cells with variant treatments did not show the obvious transformation (Figure 1). However, adding α-MSH significantly induced the cell growth when compared with the control group (Figure 2). The α-MSH-induced increase in cell counts was attenuated by treatment either with retinol or PPAR*γ* antagonist, GW9662 (Figure 2).

**Figure 3:**
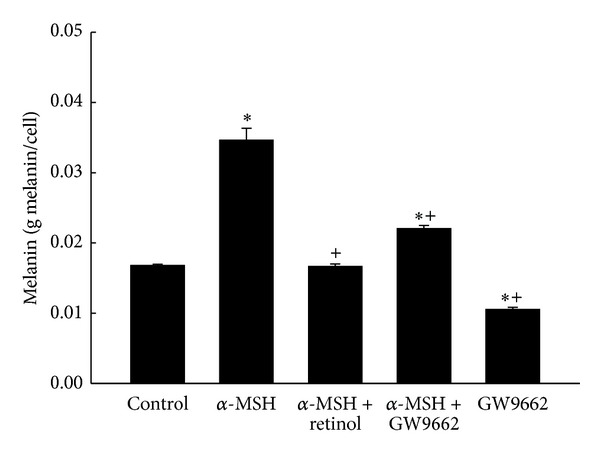
The melanin levels in five groups. Bars represent 1 SE. Significant differences compared with the control group: *P < 0.05 and **P < 0.01. ^+^Significant differences (*P* < 0.05) compared with the α-MSH group.

The results of melanin levels in groups are shown in Figure 3. The level in control group was measured as 0.017 ± 0.0002 g/mL. Treatment with α-MSH for two days significantly induced an increase in melanin level, indicating the melanogenesis. This high level caused by α-MSH reached over 2 times of the control level. That α-MSH-induced melanin synthesis was prevented completely by retinol. Adding GW9662 significantly attenuated the increase by α-MSH but still higher than that in the control group.

**Figure 4:**
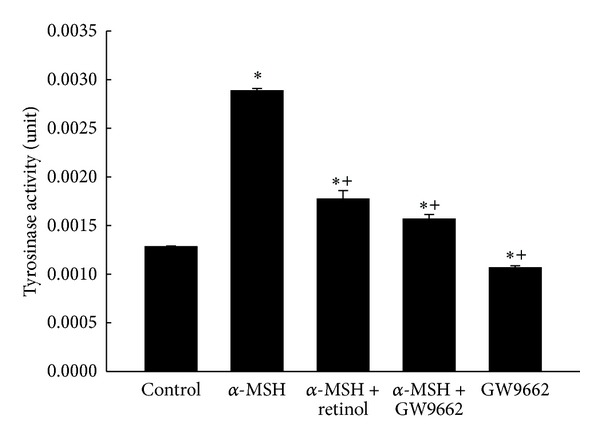
The tyrosinase activities in five groups. Bars represent 1 SE. Significant differences compared with the control group (*P < 0.05) or with the α-MSH group (^+^ *P* < 0.05).

Further analyzing the activities of tyrosinase, the key enzyme in melanogenesis in five groups is summarized in Figure 4. The results showed similar trends of melanin levels in groups. The treatment of α-MSH for two days elevated significantly the activity of tyrosinase over two times of control group. However, the α-MSH-induced increase in tyrosinase activity was prevented by retinol or by GW9662 (Figure 4).

**Figure 5:**
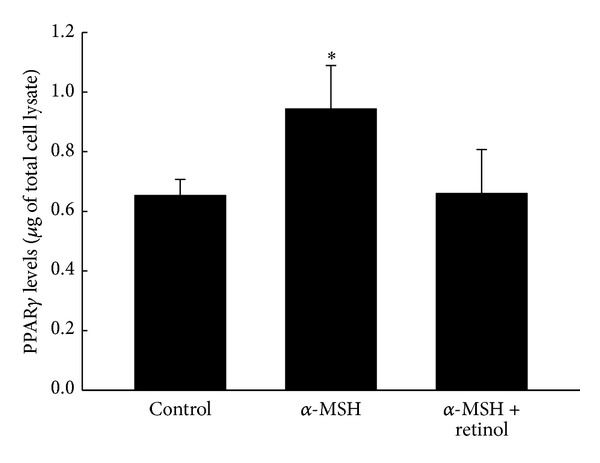
The GSH content in five groups. Bars represent 1 SE. *Significant differences (*P* < 0.05) compared with the control group.

The GSH contents in all groups are shown in Figure 5. The amount of GSH was obviously decreased by α-MSH treatment. Exogenous antioxidant, retinol, restored the GSH partially but did not reach statistical significance. Adding GW9662 to block the PPAR*γ* did not affect the decrease of GSH contents by α-MSH. However, there was no influence on the level of GSH by only GW9662 treatment when compared to the control group.

**Figure 6:**
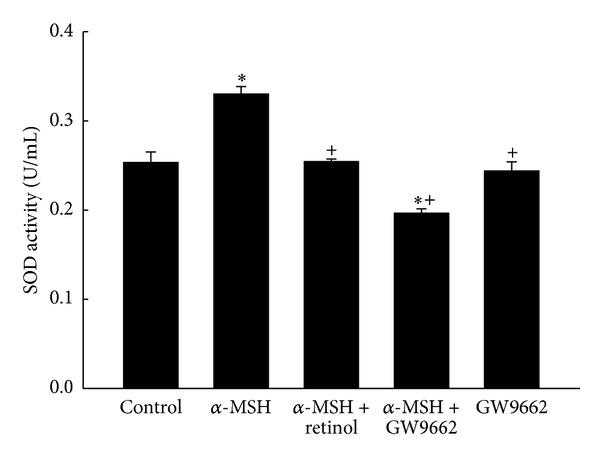
The catalase activities in five groups. Bars represent 1 SE. *Significant differences (*P* < 0.05) compared with the control group.

Figure 6 summarized the results of catalase activities in each group. The catalase activity was attenuated over 50% of control group by α-MSH. It restored significantly by coculture with retinol or GW9662 but is still lower than that in control group. Treatment of GW9662 only resulted in obvious decrease in catalase activity when compared with the control group.

**Figure 7:**
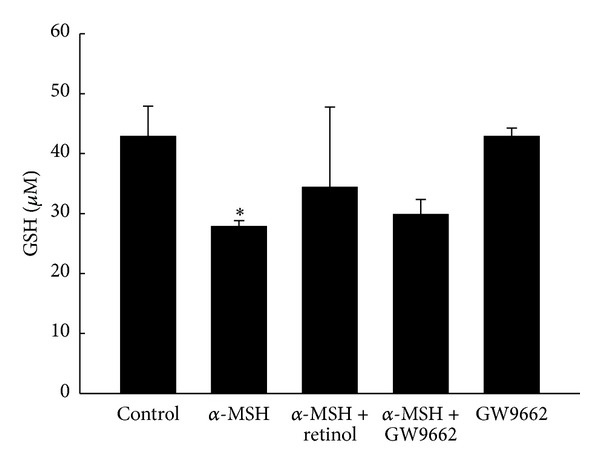
The superoxide dismutase activities in five groups. Bars represent 1 SE. *Significant differences (*P* < 0.05) compared with the control group. ^+^Significant differences (*P* < 0.05) compared with the α-MSH group.

Detecting the activities of SOD in each group was summarized in Figure 7. It was induced increase by α-MSH stimulation. The α-MSH-induced increase was prevented by retinol or GW9662. There was no difference between the control group and the group of GW9662 only.

**Figure 8:**
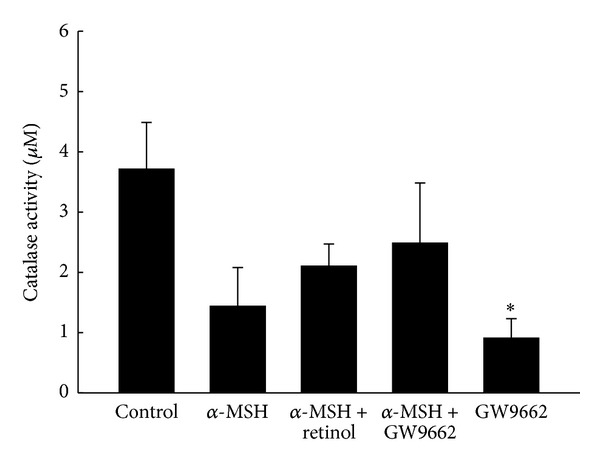
The PPAR*γ* levels in the control, α-MSH, and α-MSH+retinol groups. Bars represent 1 SE. *Significant differences (*P* < 0.05) compared with the control group.

The levels of PPAR*γ* in each group are shown in Figure 8. There was a significant increase in the PPAR*γ* level caused by adding α-MSH. Treatment of retinol prevented the increase in PPAR*γ* expression.

## 4. Discussion

Melanogenesis was demonstrated to protect against the reactive oxygen species (ROS) in many documents. We hypothesized that melanogenesis was via activation of PPAR*γ*, which elevated the tyrosinase activity. In addition, pretreatment of antioxidant retinol was able to prevent these effects. The melanogenesis was well known mediated by tyrosinase, the rate-limiting enzyme in the process of melanin synthesis. Tyrosinase converts the L-tyrosine to dopaquinone for the synthesis of both pheomelanin and eumelanin. Many stimuli to induce melanogenesis processing such as α-MSH [25, 26] and UV radiation exposure [12] were via elevating the oxidative stress. Indexes of melanogenesis, such as melanin contents, tyrosinase activity, and expression, were all augmented by α-MSH [25] and UV radiation exposure [26]. However, pretreatment of retinol significantly reduced above index of melanogenesis in a dose-dependent manner [25]. It was demonstrated that the effects about melanogenesis by α-MSH were via activating melanocortin 1 receptor (MC1R) followed by activation of the CREB/MITF pathway [11]. The protection of melanogenesis against ROS was identified in another kind of cells, primary transformed retinal pigment epithelium cell (RPEC) [27]. When MC1R was blocked either by antagonist or by si RNA, the melanogenesis-related protection was abolished. Furthermore, the melanogenesis-related survival effects on RPECs diminished by Akt inhibitor treatment. Therefore, the functions of melanogenesis were identified as Akt related pathway [27]. Not only melanogenesis but also proliferation was induced by α-MSH [25]. The proliferation index, cell numbers of B16 cell line, was elevated obviously by α-MSH treatment [25]. The cell growth induced by α-MSH (10 nM) was prevented 53% by retinol treatment at 20 *μ*M [25]. In the present study, the increases in cell counts, melanin level, and tyrosinase activity were measured by α-MSH treatment (Figures 2, 3 and 4), supported by the above documents [25, 27]. In addition, both the proliferation and the melanogenesis were reversed by pretreatment of antioxidant retinol (Figures 1, 2, and 3), which was also consistent with the results by Sato et al. [25]. Furthermore, pretreatment PPAR*γ* antagonist, GW9662, prevented the α-MSH-induced increases in melanin level (Figure 3) and tyrosinase activity (Figure 4). We further detected the marked augment in PPAR*γ* level by α-MSH treatment when it was compared with that in control group (Figure 8). Thus, we demonstrated that the melanogenesis in B16F10 cells was dependent on the activation of PPAR*γ*. It is confirmed to our hypothesis that the melanogenesis was via activation of PPAR*γ* and pretreatment of retinol abolished the process.

We hypothesized that the anti-inflammatory effects of melanogenesis depended on the activation of PPAR*γ* to trigger the activity of tyrosinase. The anti-inflammation of melanogenesis was observed in mouse models [9, 28]. UVB exposure (150 mJ/cm^2^) led to ICR-Foxn/^*nu*^ mice dorsal skin erythema, desquamation, and transdermal water loss. Immunostaining results showed an increase in the activation of cyclooxygenase-2 (COX-2) but decreased in catalase activity [9]. Both the COX-2 and the PPAR*γ* were found in melanoma by immunohistological staining [29] and activation of PPAR*γ* by rosiglitazone attenuated COX-2 expression [30]. The ROS not only directly damaged proteins, lipids, and DNA, meanwhile, the increase in intracellular ROS, especially hydroperoxide, also acted as mediators to trigger the MAPK pathway (ERK, p38 and JNK) after UVB exposure [15, 16]. Recently finding reported by Peng et al. [26] demonstrated that the ROS is able to induce increases in tyrosinase and melanin expression by JNK and ERK signaling pathway [26]. Therefore, pretreatment of soy isoflavone, having potent antioxidant activity, significantly protected ICR-Foxn/^*nu*^ mice from harms by UVB exposure [9]. Another piece of evidence was shown by Luger et al. [28]. When the UV-light exposure was carried out on mice, the release of α-MSH from keratinocytes was induced to alter the functions of antigen presenting cells and vascular endothelial cells [28]. In addition, administering α-MSH to induce melanogenesis either by intravenous or by topical application to mice inhibited the induction of hypersensitivity reaction, indicating the anti-inflammation [28]. They concluded that the anti-inflammatory effects of melanogenesis were related to the capacity to alter the functions of antigen presenting cells and vascular endothelial cells [28]. Activation of PPAR*γ* leading to anti-inflammation was well known to date. A piece of evidence which also supported our hypothesis was that the inflammation and immune responses caused by UV-light were augmented by inactivation of PPAR*γ* [31]. Acute UVB irradiation, similar to that used by Luger et al. [28], was carried out on epidermal PPAR*γ* knockout mice, Pparg (Pparg-/-(epi)), which was a species of the SKH-1 hairless albino mice [31]. The augments of UVB-induced Caspase 3/7 activity, apoptosis, and inflammation were extremely more obvious than those in wild type littermates [31]. Consistently, increases in apoptosis and inflammation were performed after blocking the PPAR*γ* activity by GW9662 treatment [31]. Above research supported our hypothesis that the anti-inflammatory effects of melanogenesis depended on the activation of PPAR*γ* to trigger the activity of tyrosinase. In our study, both the melanin level and the tyrosinase activity were elevated by α-MSH treatment when compared to those in the control group. However, these increases were attenuated by treatment of GW9662, the antagonist of PPAR*γ* (Figures 3 and 4). Furthermore, we detected the high level of PPAR*γ* in the α-MSH group. Therefore, we demonstrated that the α-MSH caused an increase in PPAR*γ* level, elevating the tyrosinase activity, which followed triggering melanogenesis.

In addition to reducing COX-2 activity and increasing tyrosinase activity, we hypothesized the α-MSH-induced PPAR*γ* increase contributed to the activities of antioxidant(s). Schmilovitz-Weiss reported that PPAR*γ* induced an increase in SOD level [32]. The ultraviolet (UV) light exposure [28] or oxidative stress [27] was known to induce the α-MSH secretion, which had capacity to trigger melanogenesis and proliferation in B16/F10 cells. Cheng et al. [27] recently reported that the α-MSH protected RPEC from hydrogen peroxide- (H_2_O_2-_) induced apoptosis, via melanocortin 1 receptor (MC1R) and the Akt dependent pathway [27]. On the other hand, the relationship between antioxidants and PPAR*γ* was performed in* in vivo* study [32]. The animal model of nonalcoholic fatty liver disease was established by fed rats with fructose-enriched diet for 5 weeks [32]. The gene expression and protein level of both SOD and PPAR*γ* were obviously lower than rats with standard rat chow diet [32]. Daily administration of rosiglitazone (10 mg/kg) during the last 2 weeks of the fructose-enriched diet significantly reversed the changes by fructose-enriched diet, including SOD gene expression and both SOD and PPAR*γ* protein levels [32]. Their results indicated that the activation of PPAR*γ* led to the synthesis of SOD [32]. Interestingly, the responses of SOD, catalase, and GSH to stimulus seemed conflicting in documents. Observations were reported by Shindo et al. [33] that antioxidants, including catalase, GSH, and SOD, in the epidermis and dermis of hairless mice diminished immediately after UV light exposure [9, 33]. However, the activities of antioxidants changed with the patterns of UV irradiation, and the recovery capacities of antioxidants altered during the acute and chronic phases [34]. When the authors irradiated human skin fibroblasts with a single exposure to UV irradiation (1, 6 or 12 J/cm^2^) and then examined the activities of antioxidant enzymes over the following days, the catalase activity was attenuated immediately, then restored on the fifth day after UV exposure, indicating adaptive antioxidant response. SOD activity decreased significantly on the third day and then was restored to normal level on the fifth day after UV exposure [34]. Another supporting observation was documented by Poswig et al. in 1999 [35]. The SOD was induced by UVA irradiation and the adaptive antioxidant response was present when repetitive UV exposure [35]. Single exposure of human dermal fibroblasts to UVA irradiation resulted in a dose- and time-dependent increase in specific SOD mRNA levels [35]. When cells are exposed to UVA of 300 kJ per m^2^ for 9 to 12 hours, the activity and the amount of SOD reached almost 180% and 200% of control, respectively [35]. Repetitive UVA exposure, especially, on the first three days at a dose rate of 200 kJ per m^2^ resulted in a 5-fold induction of SOD mRNA levels, which contributed to eliciting SOD activity [35]. In our study, the treatment of α-MSH for two days continuously significantly caused increases in the PPAR*γ* level (Figure 8) and the activity of SOD (Figure 7). The α-MSH-induced increase in the activity of SOD was prevented by GW9662 administration (Figure 7). However, the conflicting responses to the α-MSH treatment were performed in antioxidants, catalase, and GSH in the present study. The activities of both catalase and GSH decrease after α-MSH treatment though the changes did not reach the statistical significance (Figure 5). We attributed the differences among our study and others to different experiment model including study subjects, stimulus, and especially the analysis timing. In addition to PPAR*γ*, the other member of PPAR family also expressed the capacity of anti-inflammation. UVB exposure (150 mJ/cm^2^) to hairless mice 2 (HRM2) every other day for 17 days caused increases in levels of proinflammatory mediators, such as NF-kB, iNOS, and COX-2, whereas activation of PPARα by pretreatment with fenofibrate downregulated these effects of UVB exposure [30]. That indicated the potential anti-inflammation of PPARα.

In conclusion, the results presented here supported the hypothesis that melanogenesis was via activating PPAR*γ*, which also modulated the balance among antioxidants. This is the first study to perform the relationship among antioxidants, melanogenesis, and PPAR*γ*. It is necessary to carry out more studies, such as clinical studies, to understand the details in unknown mechanism. That would offer more helpful information in the treatment and usage in clinic.
